# Human Mesenchymal stem cells program macrophage plasticity by altering their metabolic status *via* a PGE_2_-dependent mechanism

**DOI:** 10.1038/srep38308

**Published:** 2016-12-02

**Authors:** Anoop Babu Vasandan, Sowmya Jahnavi, Chandanala Shashank, Priya Prasad, Anujith Kumar, S. Jyothi Prasanna

**Affiliations:** 1 School of Regenerative Medicine, Manipal University, Bangalore, 560065, India

## Abstract

Mesenchymal stem cells (MSCs) are speculated to act at macrophage-injury interfaces to mediate efficient repair. To explore this facet in-depth this study evaluates the influence of MSCs on human macrophages existing in distinct functional states. MSCs promoted macrophage differentiation, enhanced respiratory burst and potentiated microbicidal responses in naïve macrophages (Mφ). Functional attenuation of inflammatory M1 macrophages was associated with a concomitant shift towards alternatively activated M2 state in MSC-M1 co-cultures. In contrast, alternate macrophage (M2) activation was enhanced in MSC-M2 co-cultures. Elucidation of key macrophage metabolic programs in Mo/MSC, M1/MSC and M2/MSC co-cultures indicated changes in Glucose transporter1 (*GLUT1* expression/glucose uptake, IDO1 protein/activity, *SIRTUIN1* and alterations in AMPK and mTOR activity, reflecting MSC-instructed metabolic shifts. Inability of *Cox2* knockdown MSCs to attenuate M1 macrophages and their inefficiency in instructing metabolic shifts in polarized macrophages establishes a key role for MSC-secreted PGE_2_ in manipulating macrophage metabolic status and plasticity. Functional significance of MSC-mediated macrophage activation shifts was further validated on human endothelial cells prone to M1 mediated injury. In conclusion, we propose a novel role for MSC secreted factors induced at the MSC-macrophage interface in re-educating macrophages by manipulating metabolic programs in differentially polarized macrophages.

Stage-specific functional roles of macrophages ranging from injury initiation to inflammation and finally instructing repair are crucial for tissue homeostasis. Macrophages shift gears oscillating between pro-inflammatory M1 and debris-scavenging/tissue-remodeling alternatively activated M2 states. These shifts are imperative for regeneration. The role of alternatively activated macrophages has been decisively proven in setting the regenerative phase in mouse models of skin, skeletal muscle and myocardial injury^1^,^2^,^3^.

Apart from macrophages, over the past few years, *ex vivo* cultured mesenchymal stem cells (MSCs) have emerged as potent transplantable reparative cells, with astounding therapeutic benefits in animal models of disease [Reviewed by refs 4, 5, 6]. Ongoing clinical trials across the globe with human MSCs for several degenerative diseases are equally promising (refer ClinicalTrials.gov). Immuno-modulation, together with trophic factor release, has been attributed as pivotal components of MSC-mediated repair^7^,^8^,^9^. Further, inflammation and injury derived cues are essential to trigger MSC based immune-modulation^10^,^11^. One well accepted mechanism is hijacking of T cell responses towards a regulatory T cell phenotype^12^,^13^,^14^. However, chronic injury and progressive degeneration is a complex condition orchestrated by several immune players apart from T cells. Recently, there is renewed interest in macrophages due to existence of these cells in varied functionally plastic modules bridging innate and adaptive immunity^15^. Adoptive transfer of alternatively activated macrophages have conferred protection in inflammatory diseases such as Type I diabetes, indicating their decisive roles in reprogramming host inflammation^16^. Further, few very recent evidences indicate an increased recruitment of anti-inflammatory macrophages in *in vivo* models of MSC transplantation were regeneration and repair was noted^17^,^18^. Macrophages exist in a spectrum of functional states and exaggerated anti-inflammatory (M2) or inflammatory (M1) macrophage functional states are associated with distinct pathologies. Thus we hypothesize that the exact functional state of macrophage at the MSC interface could play a critical role in influencing the therapeutic outcome upon MSC transplantation. Previous evidences from mouse MSC/macrophage interaction studies indicate broad changes in M1/M2 balance during *in vitro* and *in vivo* interface^19^,^20^,^21^ However, the exact relevance of human MSC- human macrophage cross-talk when interfaced with “distinct” macrophage functional modules is underappreciated.

This study is an attempt to explore the MSC-macrophage cross-talk in depth, at each stage of macrophage activation, right from transition of monocyte to differentiated macrophage, and their activation into different effector modules in MSC-macrophage co-cultures. MSCs facilitate monocyte to macrophage transition, potentiate microbicidal responses, skew naïve macrophages to a M1 state, attenuate already activated M1 macrophages and enhance M2 activation. Mechanistic insights from the study indicate a two-way interaction between MSCs and macrophages, wherein MSC secreted factors induced at the MSC-macrophage interface instruct critical metabolic switches in M1 and M2 macrophages which are known to exist in distinct bioenergetic states. Further, the relevance of this cross-talk is discussed in context of macrophage mediated endothelial injury. Observations discussed in the manuscript raise challenging questions regarding the immune-modulatory plasticity of MSCs when interfaced with distinct macrophage activation states.

## Results

### Mesenchymal stem cells facilitate monocyte to macrophage transition

**Figure 1:**
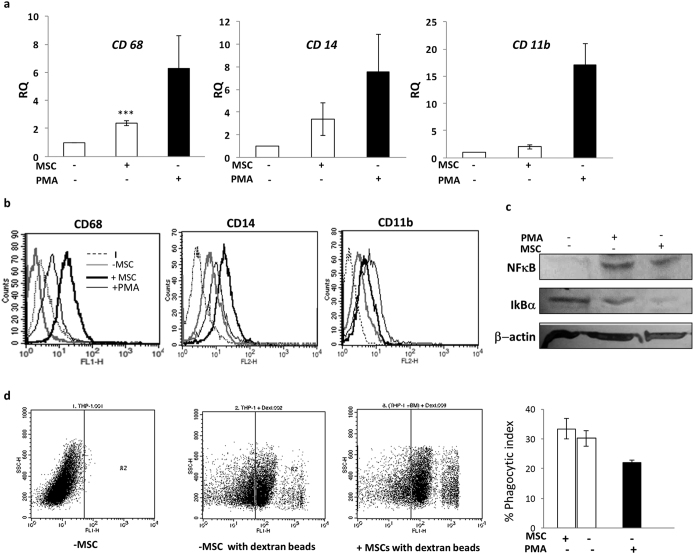
MSCs facilitate monocyte to macrophage differentiation in co-cultures. THP-1 cells were either co-cultured with MSCs (+MSC) or differentiated into macrophages with PMA (denoted as +PMA) for 48 h. MSCs were seeded in upper inserts for MSC-THP-1 co-cultures. After co-culture, cells were washed and analyzed for mRNA expression levels (**a**), flow-cytometry (**b**), western blot analysis (**c**) or phagocytosis assays (**d**). PMA treated THP-1 was used as differentiated controls for comparison in all the experiments (black filled bars). (**a**) RQ represents fold increase in human monocyte-macrophage transcripts in comparison to untreated THP-1 cells. (**b**) Flow-cytometry assessment of cell surface antigens induced upon monocyte to macrophage differentiation. Each histogram represents cell surface expression on THP-1 cells (thick grey line), PMA differentiated THP-1 cells (thin black lines) and THP-1 co-cultured with MSCs (thick black line). Dotted line represents isotype control (I) staining. Histogram overlay is representative of three independent experiments. (**c**) Representative western blot depicting proteins immune-probed towards the left. β-actin levels was used as loading control. (**d**) Dot plots represent cells with or without phagocytized dextran-FITC beads. R2 in each dot plot represent cells positive for dextran-FITC uptake. Bar graphs represents % phagocytosis index of THP-1 cells after normalizing with Cytochalasin treated controls (n > 3).

Monocytes upon sensing several environmental stresses are recruited to damaged and infected tissues and are differentiated to macrophages en-route. Human monocyte cell line, THP-1, used in this study, responds to PMA (a known PKC activator) stimulation and differentiates to adherent macrophages (Mφ) exhibiting all the bona fide markers (CD68, CD14 and CD11b) associated with human macrophages. Further, NFκB induction and activation *via* the IκB canonical pathway, marks the differentiation of monocytes to macrophages^22^. To analyze whether MSCs can modify monocyte state, MSCs were co-cultured with human monocyte line THP-1 and evaluated for induction of NFkB and macrophage associated cell surface antigens described above. Induction of CD68 and CD14 transcript and cell surface expression was noted in Mφ and MSC-THP-1 co-cultures (Fig. 1a,b). PMA activated THP-1 cells differentiated into Mφ as depicted by up-regulation of NFκB and concomitant reduction in IΚBα levels (Lane 2, Fig. 1c). Surprisingly, undifferentiated THP-1 cells exposed to MSCs, exhibit NFκB induction and reduction/degradation in cytosolic IκB suggesting activation of NFκB *via* the canonical pathway akin to PMA differentiated THP-1 cells (Lane 3, Fig. 1c). In addition, THP-1 monocytes are more phagocytic as compared to macrophages which evolve to perform more specialized antigen presenting cell (APC) functions. A decrease in phagocytic index was noted in PMA differentiated macrophages as compared to THP-1 monocytes as reported. However, MSC co-culture did not alter the phagocytic capacity of THP-1 cells (Fig. 1d). An overall analysis of undifferentiated THP-1-MSC co-cultures indicates a possible transition of naïve THP-1 monocytes to macrophage-like state upon MSC exposure.

### MSCs potentiate the innate ability of macrophages to respond to pathogen insult

Monocytes and macrophages have innate ability to curb infections at injured sites much before adaptive immunity sets in and serve as janitors of the immune system. Contact with pathogens induces respiratory burst in monocyte/macrophage lineage cells facilitating pathogen clearance mechanisms. Respiratory burst is marked by induction of NADPH oxidase subunits further leading to enhanced Reactive Oxygen Species (ROS) and up-regulation of inducible Nitric oxide synthase (iNOS)^23^. NO thus formed reacts with Reactive oxygen intermediates (ROI) to generate peroxy-nitrates or reactive nitrite intermediates (RNI) which along with ROI result in pathogen killing/reduction in pathogenic load.

**Figure 2:**
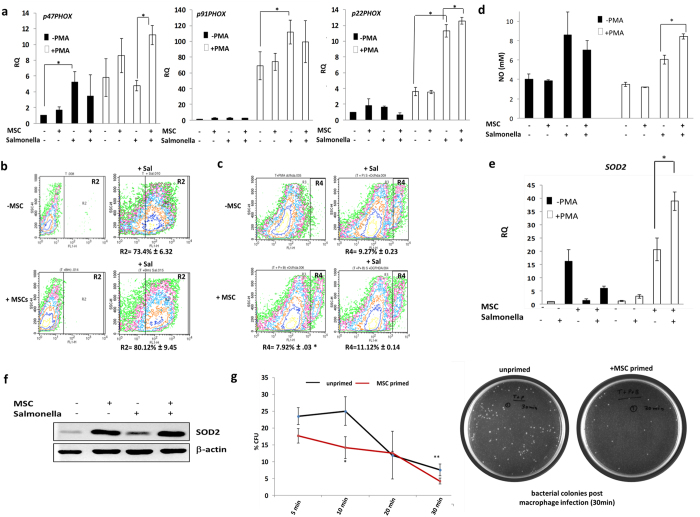
MSCs facilitate respiratory burst related genes/proteins and enhance microbicidal functions of macrophages. (**a**) Changes in mRNA expression of inducible NADPH oxidase subunits upon *Salmonella* infection. (**b**,**c**) Flow-cytometry based assessment of DCFH-DA fluorescence, indicating ROS levels in human monocytic THP-1 cells (**b**) and PMA differentiated Mφ (**c**). Gates were set based on DCFH-DA fluorescence in control untreated cells. Values below histogram represent % DCFH-DA florescence in MSC/*Salmonella* treated cells after gating with respect to untreated THP-1 or PMA differentiated Mφ as indicated by cells in regions R2 and R4 respectively. (Values represent Mean % DCFH-DA florescence ± SEM is for 3 independent isolates) (**d**) NO production measured by Griess reaction. (**e**) Fold increase in human *SOD2* mRNA expression (**f**) Immunoblot depicting SOD2 protein levels in *Salmonella* infected and uninfected Mφ or Mφ-MSC co-cultures (**g**) % CFU *Salmonella* measured by pour plate method, post-incubation with Mφ, in Mφ lysate as indicated in material and methods. CFU after plating *Salmonella* cultures (at densities equivalent to MOI 5) unexposed to macrophages were considered as 100%. RQ represents fold increase of specific mRNA in (**a,d,f**) after normalizing with expression levels in uninfected monocytic THP-1 cells (considered as 1). All experiments are indicative of at least 3 independent experiments using different MSC isolates. *Is p value < 0.05. Black bars represent fold increase in mRNA in monocytes and white bars indicate fold increase in macrophages (**a,d,f**).

THP-1 monocytes/Mφ or MSC-primed THP-1/Mφs were subjected to *Salmonella enterica* infection to study comparative respiratory burst responses. Respiratory burst was studied by evaluating the levels of inducible NADPH oxidase subunits (PHOX p47, PHOX p91 and PHOX p22) and total cellular ROS /RNI. *Salmonella* enterica infection induced *PHOX p91* and *PHOX p22* expression in naïve PMA differentiated Mφ which was unaltered or further enhanced respectively upon MSC-co-culture. *PHOX p47* induction was not noted in *Salmonella* enterica infected Mφ cultures but MSC priming potentiated *PHOX p47* induction in infected Mφs (Fig. 2a).

In concurrence with *PHOX p47* induction, *Salmonella enterica* infection induced total ROS in THP-1 monocytes. However, no further induction in ROS was noted in either uninfected or *Salmonella enterica* infected THP-1 cells upon MSC co-culture (Fig. 2b). Mφ had higher basal levels of ROS as compared to undifferentiated THP-1 cells owing to higher levels of inducible NADPH oxidase subunits (Fig. 2b,c). To our surprise, MSC co-culture further enhanced ROS levels in uninfected Mφ. However, *Salmonella enterica* infection enhanced ROS levels in both naïve as well as MSC-primed Mφ equally (Fig. 2c). Induction of anti-oxidant mechanisms like SOD2 in MSC co-cultures concomitant with induction of *PHOX* genes could explain buffering of total ROS levels in infected Mφ-MSC co-cultures (Fig. 2e,f).

NO levels were enhanced in *Salmonella enterica* infected THP-1 cells/Mφs. However, MSC co-culture resulted in enhanced NO secretion in *Salmonella enterica* infected Mφ in particular, in contrast to *Salmonella enterica* infected THP-1 monocytes where no further enhancement in NO was noted (Fig. 2d).

Though MSC primed THP-1 monocytic cells depicted few facets of differentiated Mφ, such as NFκB induction, as depicted in Fig. 1, they were not similar to true PMA differentiated Mφs with respect to respiratory burst responses.

To assess whether increase in respiratory burst responses in MSC co-cultured Mφ under infection conditions could extrapolate to enhanced microbicidal activity in Mφs, microbicidal assays were performed under co-culture conditions. MSC co-cultured Mφs could facilitate faster microbial clearance compared to control Mφs as evidenced by reduction of bacterial CFU post macrophage infection (Fig. 2g).

Thus MSCs seem to potentiate mechanisms facilitating better microbicidal functions in Mφs.

### Mesenchymal stem cells induce distinct alterations in human macrophage polarization programs depending on the activation module at macrophage interface

**Figure 3:**
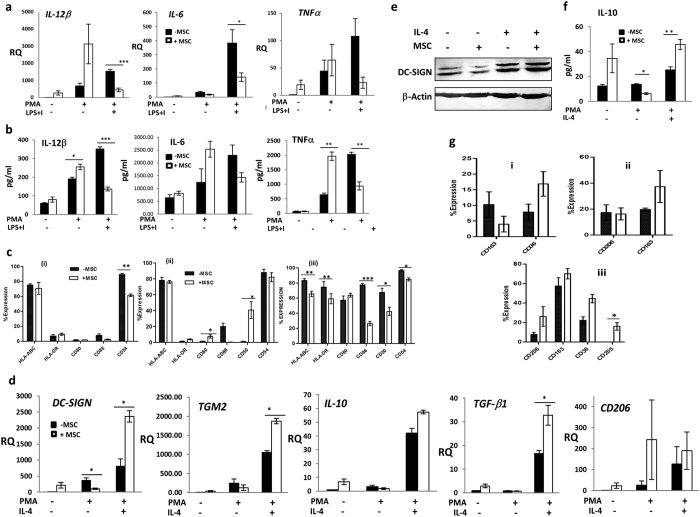
MSCs attenuate M1 activation and enhance M2 activation. **(a)** Fold increase in mRNA levels of pro-inflammatory cytokines in monocytic THP-1, PMA differentiated macrophages (Mφ) and M1-state macrophages (LPS{L} and IFNγ {Ι} stimulated THP-1) upon MSC-co-culture. **(b)** Pro-inflammatory cytokine levels in THP-1, Mφ and M1 supernatants with (white bars) or without (black bars) MSCs measured by ELISA. **(c)** % Expression of surface antigens associated with APC-capacity in THP-1 (i), Mφ (ii) and M1 activated THPs (iii) assessed by flow cytometry **(d)** Fold increase in mRNA transcripts associated with M2 state **(e)** Western blot depicting protein levels of DC-SIGN. Lane 1 and 2 indicate DC-SIGN in Mφ in presence and absence of MSCs respectively. Lane 3 and 4 denote DC-SIGN in M2 macrophages in presence and absence of MSCs respectively. **(f)** IL-10 levels of THP-1, PMA differentiated macrophages (Mφ) or M2 macrophages with (white bars) or without (black bars) MSCs measured by ELISA. **(g)** % Surface expression of scavenger receptors analyzed by flow cytometry on THP-1 (i), Mφ (ii) and M2 activated THP-1 (iii). Fold increase in mRNA transcripts associated with M1 (**a**) or M2 (**d**) states were obtained after normalizing with mRNA levels in THP-1 and denoted as RQ (relative co-efficient) n ≥ 3.

**Figure 4:**
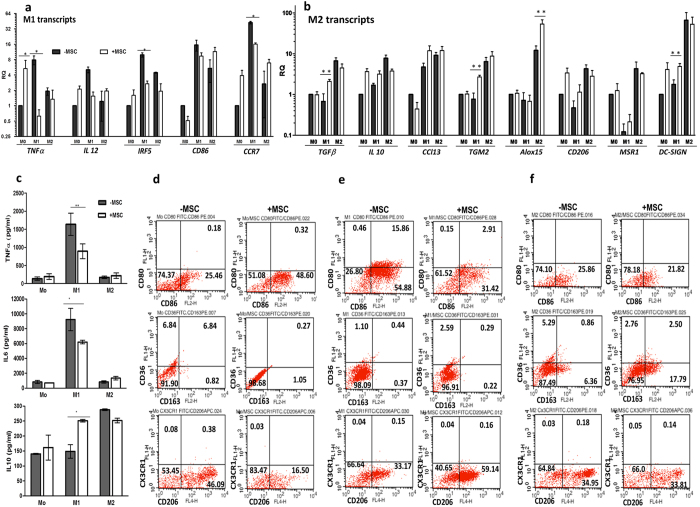
MSCs modulate human primary macrophage differentiation and activation states. (**a,b**) Fold increase in prototypic mRNA transcripts associated with inflammatory macrophages, M1 (**a**) and alternatively activated macrophages, M2 (**b**) upon co-cultures of naïve macrophages (Mo), M1 polarized (M1) or M2 polarized macrophages (M2) with MSCs. RQ was obtained after normalizing with mRNA levels in naïve primary macrophages (n = 3, Mean ± SEM). (**c**) Cytokine levels of TNFα, IL6 and IL10 in supernatants of naïve macrophages (Mo), M1 and M2 macrophages with (white bars) or without (black bars) MSC co-culture (n = 3). (**d,e,f**) Representative flow cytometry assessment of CD80/CD86, CD36/CD163 and CX3CR1/CD206 expressing cell populations in naïve Mo (**d**), M1 polarized (**e**) and M2 polarized macrophages (**f**) with (+MSC) or without MSC (−MSC) co-culture. Values in each quadrant of the dot plots depict mean % cells in each population (either double positive, single positive or double negative for the combination of cell surface antigens analyzed).

To evaluate how MSCs modulate M1 or M2 polarized macrophages, MSCs were co-cultured with either PMA differentiated THP-1 cells or primary human macrophages (differentiated from peripheral blood mononuclear cells by human macrophage colony stimulating factor, M-CSF) and activated by pro-inflammatory stimuli, LPS and IFNγ (M1) and alternative stimuli, IL-4 (M2) respectively. We also evaluated M1 and M2 skewing in naïve PMA differentiated Mφ as well as M-CSF induced naïve primary macrophage (Mo) -MSC co-cultures in absence of M1 or M2 stimuli to check if MSCs alone caused specific polarization shifts (Figs 3 and 4).

Reduction in secretion and expression levels of M1 cytokines; IL-12, IL-6 and TNFα, was observed in inflammatory M1 macrophages upon co-culture with MSCs. Surprisingly, significant enhancement in release/synthesis of pro-inflammatory cytokines; TNFα and IL-12 was observed in naive Mφ upon MSC co-culture (p value < 0.05) suggesting a possible skewing towards an inflammatory M1 state (Fig. 3a,b). M1 polarized macrophages can power steer adaptive immune responses by rendering antigen presentation (APC) support to infiltrating T cells thus exaggerating inflammation. Thus the expression of surface ligands involved in APC function; MSC class I and class II ligands, key co-stimulatory molecules (CD80/CD86) and immune adhesive ligands relevant at the APC-T cell synapse; CD50 and CD54 were quantitated in MSC-Macrophage co-cultures. As expected, transition to a M1 state resulted in up-regulation of MHC class II molecules with a concomitant increase in potent co-stimulatory ligand, CD86 and CD50/CD54 (Supplementary Figure 1 and Fig. 3cii/iii). M1-MSC co-culture resulted in reduction in expression of all the above cell surface antigens on M1 activated THP-1 cells suggesting an attenuation of APC capacity of M1 macrophages (Fig. 3c). Naïve Mφ expressed lower surface levels of MHC class II and CD86 as compared to inflammatory M1 macrophages as reported. However, MSCs did not majorly modulate the levels of MHC class I or class II molecules as well as immuno-adhesive ligands in naïve PMA differentiated THP-1 Mφ co-cultures with the exception of CD86 which was down-modulated (Supplementary Figure S1ii). To further probe whether M1 attenuation in presence of MSCs is associated with switch towards an immune-suppressive M2 activation module, we checked IRF4 transcript levels in M1-MSC co-cultures. IRF4 expression levels are critical for M2 polarization^24^. Increase in *IRF4* levels, in M1-MSC co-cultures and decrease in *IRF4* in naïve Mφ-MSC co-cultures as compared to untreated M1 or Mφ cultures respectively indicated opposing polarization instructions in naïve and M1 activated macrophages by MSCs (Supplementary Figure S2).

Once infection is under check, by M1 macrophages along with recruitment of other adaptive immune components at injured sites, tissue hemostasis is eventually restored by a functional shift of macrophages to a M2 state, which sets in a phase of injury resolution, matrix remodeling and regeneration. Injury resolving and tissue remodeling-M2 macrophages are marked by high expression of scavenger and mannose receptors and by secretion of anti-inflammatory cytokines such as IL-10 and TGF-β^25^. M2 polarizing stimuli induced M2 macrophage associated transcriptional responses like *trans-glutaminase 2* (*TGM2)*, *DC-SIGN, IL-10* and *TGFβ* in PMA differentiated THP-1 cells. The expression levels of all these genes were further exaggerated in MSC-M2 co-cultures (Fig. 3D). Despite the marked induction of M2 transcripts by M2 polarizing cytokines, very low surface levels of scavenger receptors; CD205, CD163, CD206 and CD36 were noted in M2 activated macrophages under our experimental conditions in THP-1 cells. However, MSC co-culture resulted in enhanced surface expression of all the scavenger receptors studied on M2 activated macrophages (Fig. 3g, Supplementary Figure S3). Induction in M2 activation thresholds by MSCs was further validated by enhanced expression of DC-SIGN in M2 macrophages in co-culture with MSCs (Fig. 3e). Since no increase in M2 transcripts or scavenger receptors was observed in MSC-Mφ co-cultures, MSCs did not polarize naïve Mφ to a M2 state. In fact, a reduction in basal levels of DC-SIGN, *TGM2* and IL-10 secretion was noted in naïve Mφ upon MSC co-cultures (Fig. 3d,e,f).

PMA differentiation of THP-1 cells is reported to bias THP-1 macrophages to M1 polarization which explains the weak M2 polarization program in THP-1 cells. Thus M1-M2 paradigm shifts were further explored in primary human macrophages where the M1/M2 subsets are more clearly defined and M-CSF induced differentiation does not bias naïve Mo macrophages to M1 polarization. To refine and authenticate the observations on macrophage stage specific manipulations induced by MSCs in THP-1 model we investigated the expression of “both M1 as well as M2 transcriptional programs” in primary macrophages at Mo (naïve), M1 or M2 polarized states (Fig. 4a). In concurrence to the results in THP-1 model a pronounced reduction in a range of M1 transcripts and pro-inflammatory cytokines, TNFα/1L-6, was observed in M1 polarized macrophages. Interestingly, a range of human specific M2 transcripts and anti-inflammatory IL-10 was induced in primary M1/MSC co-cultures even under M1 polarized conditions indicating a MSC induced shift to a M2 state despite the absence of M2 polarizing stimuli (Fig. 4a–c).

Flow cytometry analysis of CD80/CD86 expressing macrophage populations in Mo, M1 and M2 polarized primary macrophages (Fig. 4d–f respectively) indicated an increase in CD86 expressing cells in Mo/MSC co-cultures in contrast to M1/MSC co-cultures were marked reduction in both CD86 as well as CD80/CD86 co-expressing macrophages were noted. M2 macrophages did not express CD86 and expressed low levels of CD80 in both control and co-culture conditions. M-CSF induced primary macrophage differentiation results in induction of M2 associated CD206 in naïve macrophages in contrast to PMA induced differentiation in THP-1 cells. However, MSC co-culture resulted in decrease in CD206 population in Mo state (Fig. 4d) which is in consensus with MSCs skewing naive macrophages to M1 like program as observed in THP-1 cells. CD206 expressing population was substantially increased in M1/MSC co-cultures (Fig. 4e). In a nutshell, MSCs seem to specifically skew naïve macrophages to a M1 state, attenuate M1 macrophages by shifting them to a M2 state and moderately enhance M2 activation macrophage thresholds.

### MSCs modulate IDO levels in distinctly polarized macrophages in an activation stage dependent manner

Indoleamine 2,3-dioxygenase (IDO) activity in APCs results in reduction of local tryptophan concentration facilitating suppression of T effector responses. IDO levels in macrophages correlated with polarization switches in differentiated macrophages^26^. Thus, macrophages in different activation states were assessed for IDO transcript and protein levels in MSC co-cultures to further substantiate the M1 to M2 shift in polarization spectrum.

**Figure 5:**
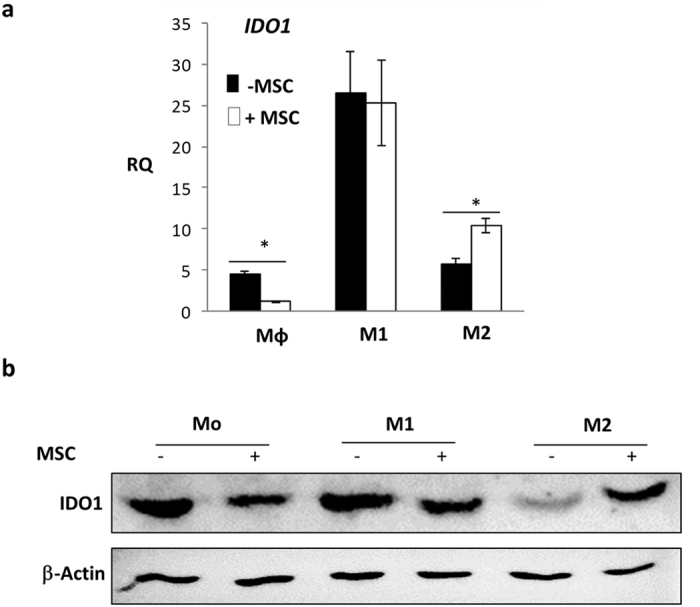
MSCs manipulate IDO levels during primary macrophage polarization shifts. (**a**) Relative mRNA levels of *IDO1* in naïve, M1 and M2 macrophages with (white bar) and without (black bars) MSC co-culture. Fold increase (RQ) is obtained after normalizing with levels in naïve PMA differentiated THP-1 cells (considered as 1). (**b**) Immunoblot depicting IDO1 protein levels in primary human macrophages existing in different polarized states in presence (+) and absence (−) of MSC as indicated on top of the blot. β-actin staining is used as a loading control.

PMA differentiated THP-1 macrophages expressed basal levels of IDO1 and attenuation of *IDO1* transcript was noted upon MSC co-culture (Fig. 5a). Absolute levels of IDO are critical for polarization choice in differentiated macrophages. High induction of *IDO1* mRNA was noted upon polarization of THP-1 cells to an inflammatory phenotype [Fig. 5a] as reported previously^26^. MSCs attenuated IDO1 transcript levels in PMA differentiated THP-1 cells and IDO1 protein levels in naïve primary human macrophages (Mo) in MSC co-cultures. A marked reduction in IDO1 levels were observed in M1 polarized primary macrophages in MSC co-cultures [Fig. 5b]. Primary M2 macrophages expressed lower IDO1 levels than M1 macrophages. Surprisingly, MSC co-culture resulted in enhanced IDO1 in M2 polarized cells. IDO feedback regulatory loops control macrophage activation modules^26^. Contrasting fluctuations in IDO1 protein in primary M1- or M2-MSC co-cultures further strengthened the possibility of distinct stage specific manipulation of macrophage activation modules by MSC and opened up a possibility of MSCs manipulating macrophage metabolic states as enhanced IDO activity modulates Tryptophan availability in cells.

### MSCs impact the metabolic phenotype of primary macrophages in co-cultures

**Figure 6:**
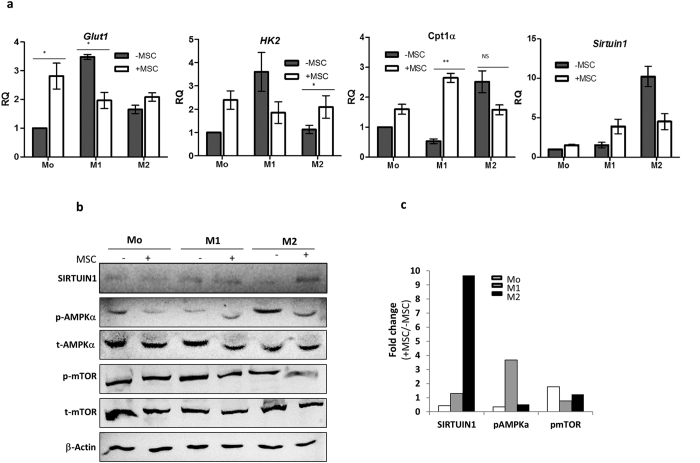
MSCs manipulate metabolic programs in primary macrophages. (**a**) Relative mRNA levels of key metabolic genes in naïve (Mo), inflammatory M1 and alternatively activated M2 macrophages in MSC co-cultures. RQ was calculated relative to levels in naïve macrophages (considered as 1). Data represents Mean ± SEM (n ≥ 3). (**b**) Immunoblot analysis depicting the protein levels of critical metabolic sensors modulated during macrophage polarization and activation in unexposed (−MSC) and MSC exposed (+MSC) macrophages. β-actin staining is used as a loading control. (**c**) Bar graphs depict mean fold change in protein levels of metabolic sensors in macrophages exposed to MSCs (+MSC) relative to protein levels in control macrophages unexposed to MSCs (−MSCs). β-actin was used to normalize protein levels across conditions. Active (phosphorylated) p-AMPKα and (phosphorylated) p-mTOR levels were first normalized with total t-AMPKα and t-mTOR respectively subsequent to normalizing with β-actin loading control. NS is not significant.

Recent insights from macrophage biology and function highlight the significance of reversible metabolic programs in influencing the functional spectrum of macrophage polarization phenotypes [Reviewed in ref. 27]. Activation of macrophages with inflammatory stimuli has been shown to cause a metabolic shift marked by increased glucose uptake and shift to glycolysis form oxidative phosphorylation analogous to Warburg’s effect in tumor microenvironments. Regulating these switches are key metabolic sensors like AMPK, mTOR and SIRTUIN1 which integrate external micro environmental signals like amino acid availability/nutrient deprivation and inflammation to macrophage polarization switches^28^. As expected primary M1 macrophages exist in a high energy demanding state and thus express greater levels of glucose transporter 1 (*GLUT1)* and glycolytic enzymes such as Hexokinase 2 *(HK2)* [Fig. 6a]. In contrast, the alternatively activated M2 polarized state exhibits a low cellular energy state, marked by inhibition of anabolic pathways and enhancement of catabolic pathways such as β-oxidation of fatty acids geared by higher AMPK activation (a sensor of low ATP levels)^28^. Higher expression of Carnitine palmitoyl trasferase1α (*CPT1α*), a rate limiting enzyme for mitochondrial β-oxidation and p-AMPKα was noted in M2 macrophages as expected [Fig. 6a,b]. Intriguingly, MSC co-culture with naïve Mo primary macrophages enhanced *GLUT1*, *HK2* levels in concurrence to M1 skewing of naïve macrophages in MSC co-cultures. In contrast, reduced *GLUT1/HK2* concomitant with increased *CPT1α* was noted in M1-MSC co-cultures. mTOR activity, in contrast to AMPK activity, helps in maintaining high energy demands in metabolically active cells such as LPS-stimulated DCs by enhancing glycolytic enzymes and glucose uptake^29^. MSCs enhanced p-AMPKα (phosphorylated AMPK) levels in M1 cells and reduction in p-mTOR (phosphorylated mTOR) was observed in macrophages from both M1- as well as M2-MSC co-cultures, further solidifying the MSC-induced metabolic shifts in macrophages from high energy demanding to a low bioenergetics state [Fig. 6b,c]. SIRTUIN1, a target of p-AMPK, is involved in inhibiting inflammation by deacetylating p65 subunit of NFκΒ^30^. As expected *SIRTUIN1* transcripts were higher in M2 macrophages and a further induction in protein levels were observed in M2 macrophages obtained from M2-MSC co-cultures [Fig. 6a–c]. In conclusion, MSCs seem to alter the metabolic status of inflammatory M1 macrophages by shifting it to a M2 like bioenergetic state.

### COX2-dependent production of PGE_2_ in MSC secretome is a major paracrine mediator instructing M1 to M2 functional- and bioenergetics shifts in co-cultures

**Figure 7:**
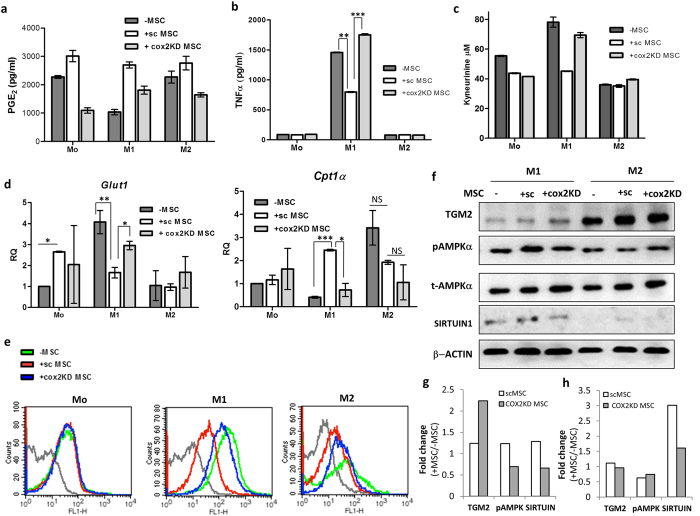
MSC secreted PGE_2_ mediates macrophage polarization shifts through metabolic reprogramming of macrophages. Mo, M1 or M2 macrophages were either unexposed or co-cultured with scrambled PKLO (vector) transduced MSCs (denoted by + sc MSC) or pKLO-*Cox2* shRNA transduced MSCs (denoted by + Cox2KD MSC) in all experiments described in this figure. (**a**) PGE_2_ levels in macrophage-MSC co-cultures quantified by ELISA and supernatants were collected for PGE_2_ analysis. Data is representative of two independent experiments each experiment performed in replicates. Values are Mean ± S.D (**b**) TNFα levels in Mo, M1 and M2 cultures exposed or unexposed (−MSC) to differentially modified MSCs as described above. Data represents Mean ± SEM (n = 3) (**c**) IDO activity reflected by Kynurenine levels in MSC-macrophage co-cultures. Data depicts IDO activity (Mean ± S.D) in a representative experiment performed in triplicates (**d**) Relative mRNA levels of *GLUT1* and *CPT1α* in Mo, M1 or M2 macrophages either exposed or unexposed (−MSC) to MSCs. Either Vector transduced or COX2shRNA vector transduced MSCs were used for co-culture with macrophages before transcript analysis in macrophages. RQ was calculated after normalizing with naïve Mo macrophages which was considered as 1. Data represents Mean ± SEM (N = 3) (**e**) Representative histogram overlay depicts comparative flow-cytometry assessment of 2-NBDG fluorescence in control Mo, M1 or M2 macrophages *versus* 2-NBDG florescence in macrophages co-cultured with scMSCs or *Cox2*KDMSCs as indicated in the panels. (**f**) Immuno-blot analysis of specific proteins in macrophages co-cultured with differentially manipulated MSCs as indicated across each panel. (**g**,**h**) Fold change in specific proteins in M1 (**g**) and M2 (**h**) macrophages exposed to either scMSCs or *Cox2*KDMSCs relative to protein levels in control macrophages unexposed to MSCs (−MSCs). β-actin was used to normalize protein levels across conditions. Active (phosphorylated) p-AMPKα were first normalized with total t-AMPKα subsequent to normalizing with β-actin loading control. Wherever depicted, p values were calculated by comparison between untreated macrophages *versus* scMSC co-cultured macrophages or alternatively comparisons were made between scMSC co-cultured macrophages and *Cox2*KDMSC co-cultured macrophages using 2-tailed unpaired students-t test. NS is not significant.

Several groups have indicated the presence of anti-inflammatory mediators such as PGE_2_ in the induced MSC secretome^31^,^32^,^33^. PGE_2_ is generated from arachidonic derivatives through a *COX2*-dependent Prostaglandin E synthase. In order to explore the role of COX2 –PGE_2_ pathways in MSC mediated macrophage reprogramming, *COX2* was knocked down in MSCs using specific shRNA (*COX2*KD MSCs) and reduction in PGE_2_ was authenticated in IFNγ primed *COX2*KD MSC culture supernatants (Supplementary Figure 4). Higher amounts of PGE_2_ was detected in M1-scrambled shRNA vector transduced MSC (sc MSC) co-cultures as compared to M1 cultures suggesting induction of PGE_2_ at the M1-MSC interface. Co-culture of *COX2*KD MSC with M1 macrophages attenuated this increase emphasizing the contribution of MSC generated PGE_2_ in the hike of PGE_2_ levels in co-cultures [Fig. 7a]. *COX2*KD MSCs were incapable of attenuating the prototypic pro-inflammatory M1 cytokine, TNFα (scMSC co-cultured M1 macrophages *versus COX2KD* MSC co-cultured M1 macrophages, p value < 0.001) and IDO activity in M1-MSC co-cultures [Fig. 7b,c]. *COX2*KD MSC were also inefficient in inducing M2 metabolic phenotype switch in M1 co-cultures. M1 macrophages from *COX2*KD MSC co-cultures expressed higher *GLUT1*, lower *CPT1*α compared to M1-scMSC co-cultures (*Glut1* levels in *sc*MSC co-cultured M1 *versus COX2KD*MSC co-cultured M1 macrophages p value < 0.05, *Cpt1α* levels in scMSC co-cultured M1 *versus COX2KD*MSC co-cultured M1 macrophages p value < 0.05) and glucose uptake similar to untreated M1 polarized macrophages [Fig. 7d,e]. However, no significant change in both *Glut1* and *Cpt1α* levels were noted in M2 macrophages when co-cultured with either scMSCs or *Cox2KD*MSCs. Further, Sirtuin1 and p-AMPKα levels were lower in M1 macrophages from M1-COX2KD MSC co-cultures as compared to M1-scMSC co-cultures [Fig. 7f,g]. However, an unexplainable increase in M2 polarization stimuli induced TGM2 was noted in *COX2*KD MSC-M2 co-cultures. Taken together the above observations indicate a critical role for COX2 pathway generated PGE_2_ from MSC in instructing macrophage polarization/metabolic shifts specifically at the M1-MSC interface.

### MSC-primed M1 macrophages are inept in causing inflammatory damage to endothelial cells

**Figure 8:**
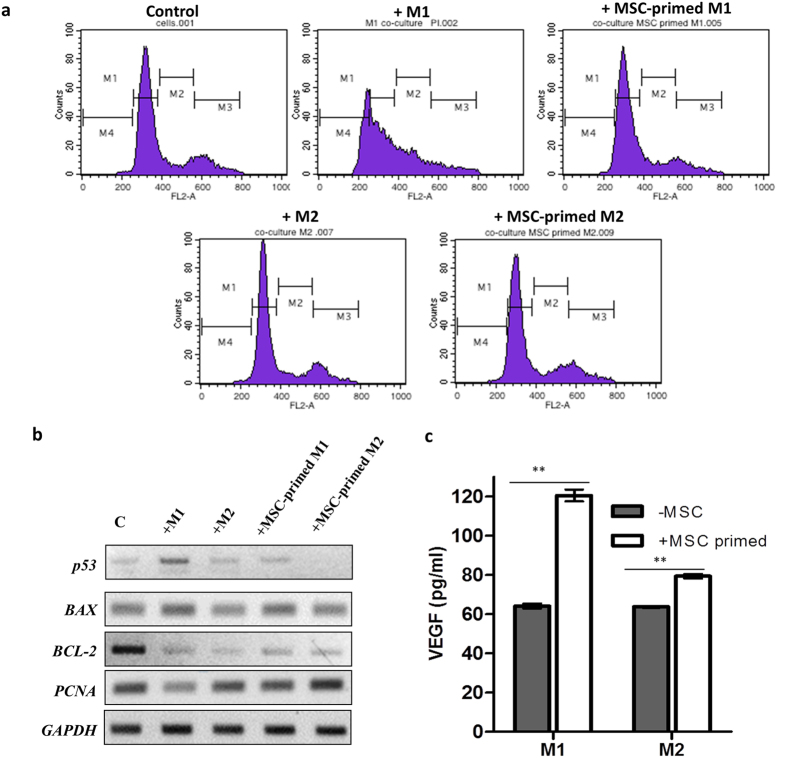
MSCs attenuate M1 mediated endothelial injury. (**a**) Cell cycle analysis of HUVEC cells co-cultured with M1 or M2 macrophages or alternatively with MSC primed M1 and M2 macrophages as indicated above each histogram. M1 represents the G_0_ phase, M2 represents the S phase, M3 represents the G2/M phase and M4 represents the sub-apoptotic phase. Gates were set with respect to control HUVEC cells and duplicated for other co-culture conditions. Figure is representative of 3 independent experiments with different MSC isolates (**b**) mRNA levels of cell cycle related transcripts in HUVEC cells upon co-culture with M1, M2 or MSC-primed-M1/M2 macrophages analyzed on a 1% Agarose gel under non-saturating PCR conditions (25 cycles). Macrophages were separated from HUVECs with culture inserts and RNA was isolated from the bottom well containing HUVECs. (**c**) VEGF levels in M1 and M2 macrophages either unprimed (−MSC) or primed (+MSC) with MSCs. Data represents Mean ± SEM (n = 3).

Inflammatory macrophages are capable of affecting endothelial function and survival and are associated with vasculopathy in chronic inflammatory disorders such as diabetes. On the other hand, M2 macrophages support endothelial function and neo-angiogenesis^34^,^35^. Thus, to validate the attenuation of M1 activation and accentuation of M2 phenotype by MSCs, endothelial survival and cycling was monitored in primary human endothelial cells (HUVECs) co-cultured with MSC-programmed M1 or M2 macrophages. As expected, co-culture with inflammatory M1 macrophages resulted in apoptosis and reduced cycling of HUVECs. Interestingly, MSC primed M1 macrophages did not cause endothelial cell apoptosis. In fact, the cycling parameters of HUVECs co-cultured with MSC primed M1 macrophages were similar to that of control untreated cells (Fig. 8a and Table 1). Co-culture with M2 macrophages did not alter majorly the cycling parameters of HUVECs. However, an increase in S phase was noted in HUVECs co-cultured with MSC primed M2 cells further supporting enhancement of M2 activation when primed with MSCs (Table 1). mRNA analysis of HUVECs with macrophage co-culture depicted a decrease in proliferation and survival related genes, *PCNA* and *BCL-2* and an increase in pro-apototic genes *BAX* and *p53* upon M1 co-culture. However, the transcript levels of *PCNA*, *BCL-2*, *BAX* and *p53* in HUVECs co-cultured with MSC-primed M1 cells were comparable to unexposed HUVECs (Fig. 8b). Interestingly, p53 levels dropped below the levels observed in control HUVECs upon co-culture with MSC primed M2 macrophages. *PCNA* levels were also marginally higher in MSC-primed M2 co-cultures. This was corroborated with enhanced S phase observed in HUVECs exposed to MSC primed M2 cells beyond control HUVECs. p53 stabilization is known to enhance arrest/apoptosis in cells, it remains to be explored whether *p53* reduction is responsible for enhanced cycling in MSC-primed M2-HUVEC co-cultures. These results further functionally validate MSC based M1 attenuation and enhanced M2 activation in context of macrophage based endothelial injury. Interestingly, MSC primed M1 cells secrete higher levels of endothelial cell protective VEGF which could possibly rescue endothelial cell growth arrest/apoptosis [Fig. 8c].

## Discussion

In the past decade, *ex vivo* cultured allogeneic MSCs emerged as key transplantation compatible cellular immune-modulators. However, how MSCs interface with a complex immune-cell network at disease micro-environments is confounding. Apart from MSCs, macrophages have emerged as interesting cell types in regenerative contexts owing to their broad functional plasticity which encompasses roles beyond immunity and host defense. They exist in several functional modules tailored by site specific micro-environmental cues. Interestingly, the functional polarization of macrophages into M1 (pro-inflammatory) and M2 (alternatively activated/anti-inflammatory/tissue remodeling) states is a T cell independent process as demonstrated in T cell deficient immuno-compromised mice^36^. Thus, macrophages are the actual drivers of both regenerative and degenerative processes depending on their functional state irrespective of adaptive immune cell contributions. In fact, few reports suggest the engulfing of allogeneic MSCs by tissue draining macrophages which result in the convertion of these macrophages into regulatory macrophages attenuating T cell responses *in vivo*^37^,^38^. Thus, manipulation of macrophage plasticity could be one of the underlying processes behind the miraculous MSC mediated reparative benefits in inflammatory contexts. Studies involving MSC transplantation in mouse models of macrophage hyperactivity, such as sepsis and zymosan induced peritonitis, disease amelioration with concomitant macrophage reprogramming was observed^39^,^40^. Similarly, MSC transplantation in a rat model of diabetes resulted in conversion of adipose tissue associated macrophages to a M2 phenotype alongside correction of other disease parameters^18^. In all the above models, MSCs possibly interacted with inflammatory macrophages which were predominant in the disease scenario. Further co-culture of mouse peritoneal macrophages with adipose derived MSC *ex vivo* and transplantation of these MSC educated macrophages ameliorated LPS induced systemic inflammation in mice. Though secretion of anti-inflammatory mediators such as IL-10 by MSC-educated macrophages has been proposed to mediate this effect the exact mechanisms of how MSCs educate macrophages to attenuate inflammation and facilitate repair is not understood^21^,^41^.

As macrophages exist in spectrum of functional modules in different disease contexts (for *eg.,* M2 macrophages are the predominant sub-type in context of fibrosis and tumors), a stage specific interaction study between MSCs and monocytes/macrophages is required. Further, most studies relied on a mouse immune cell/ human MSC interface in a complex *in vivo* scenario where macrophage attenuation could be a bystander effect of a broad MSC mediated immune-suppression. Species specific differences in macrophage biology, non-functionality of growth factors and paracrine modulators at the mouse-human interface would result in inappropriate assessment.

Thus, the present study is a novel in depth attempt to explore human MSC- human macrophage interaction in a “stage specific fashion” right from monocyte to macrophage differentiation to polarization into specific functional modules to address the above lacunae in understanding the mechanisms existent at the MSC-macrophage interface. Since the innate function of macrophages is host defense against infection, we attempted to evaluate anti-microbicidal responses to *Salmonella enterica* infection in macrophage-MSC co-cultures, a previously unexplored aspect. MSCs induced respiratory burst and NO dependent killing mechanisms in macrophages, which facilitated better microbial clearance by macrophages in MSC co-cultures (Fig. 2). This aspect could confer protection against infection in MSC transplant scenarios and also potentiate better healing. Recently, adipose derived mouse MSCs were shown to manipulate mouse macrophages obtained from a mouse model of *Leishmania* infection. MSC-educated macrophages were shown to exhibit lower TNFα/ IL-10 ratios; however the relevance of these observations to actual microbicidal functions of macrophages was not evaluated^20^. MSCs seem to sense macrophage activation thresholds/states; MSCs tend to skew naïve macrophages to a pro-inflammatory M1 polarized state as evidenced by enhanced TNFα expression and secretion (Figs 3b and 4a) and reduction in levels of M2 associated IL-10 and DC-SIGN levels (Fig. 3e,f). This also explains the enhanced capacity of macrophages to elicit microbicidal functions in MSC co-cultures, as MSC primed naïve macrophages adopt a pro-inflammatory profile beneficial in an infection situation. Co-culture of naïve primary macrophages with MSCs resulted in enhanced expression of CD86 indicating a probable increase in co-stimulatory functions as well as shift to a M1 like state [Fig. 4c]. Co-culture with activated M1 macrophages resulted in robust attenuation of M1 state as indicated by marked reduction in pro-inflammatory cytokine expression and secretion and attenuation of co-stimulatory molecules and ligands relevant for T cell activation at the APC-T cell synaptic junction (Figs 3a–c and 4c,d). Exosomes from Umbilical cord derived MSCs has previously been reported to attenuate inflammatory THP-1 cells (42). In stark contrast, MSCs further enhanced the activation of M2 functional states as indicated by consistent induction of M2 related gene programs, enhanced secretion of immune-suppressive IL-10 and scavenger receptor expression under M2 activation conditions in the THP-1 macrophage model (Fig. 3d–g). Induction of M2 gene programs concomitant with reduction of M1 genes/ co-stimulatory ligands CD80/CD86 and up regulation of scavenger receptors such as CD206 in human M1 primary macrophages indicate a M1 to M2 shift in inflammatory macrophages upon MSC priming [Fig. 4]. M2 activation module was not exaggerated in primary M2-MSC co-cultures which was in contrast to observations in the THP-1 monocyte cell line model; however MSC co-culture did not result in a M2 to M1 shift thus indicating maintenance of the M2 phenotype even under co-culture conditions [Fig. 4a–d]. When gingival MSCs were transplanted in a wound model, MSCs were spatially associated with wound macrophages with a M2 phenotype as compared to control mice where a predominance of M1 macrophages were noted^43^. However, it was not clear whether the existing M1 cells were reprogrammed to a M2 state or M2 cells were recruited at the wound interface upon MSC transplantation. The simplicity of the *in vitro* human primary macrophage model used in this study permits this assessment. Further the mouse macrophage-MSC interface could be very different from the human macrophage-human MSC interface due to key differences in macrophage biology in the human and mouse system^44^. Though MSC-educated peritoneal macrophages alleviated systemic inflammatory responses in LPS induced inflammation model, a parallel study to establish a similar concept in human macrophages from Acute respiratory distress syndrome (ARDS) did not exactly mimic mouse macrophage observations^21^. In a clinical trial with Adipose derived MSCs for correction of ARDS, an improvement of disease parameters were noted upon MSC transplantation. However no significant increase in M2 polarization was noted upon culture of patient macrophages with ARDS serum obtained from either control or MSC transplanted subjects indicating that human MSC could decipher unique programming instructions to human macrophages distinct from those existent at the mouse MSC/Macrophage interface^21^. Further distinct paracrine factors from different tissue specific MSC could influence immune outcomes in co-culture and transplant settings. Human placental MSCs were shown to exhibit similar M1 to M2 shifts in *in vitro* co-culture settings as that of bone marrow derived MSCs^45^. However, conditioned medium (CM) derived from MSCs generated from iPSC (iPSC-MSCs) could ameliorate cardiomyopathy better than CM derived from adult bone marrow derived MSC. iPSC-MSC CM was richer in macrophage migration inhibitory factor in comparison to bone marrow derived MSC conditioned media which could explain lower fibrotic and ROS responses^46^. Thus, different tissue derived MSCs could drive divergent macrophage polarization programs owing to differences in their secretomes.

Increasing evidences suggest the importance of metabolic processes in controlling macrophage polarization modules [Reviewed in refs 27 and 28]. M2 macrophages exist in a low energy demanding states in contrast to M1 macrophages where Glut1 mediated glucose uptake and accelerated glycolytic inputs established energy demanding inflammatory phenotypes^47^. MSC-educated M1 macrophages exhibited reduction in pro-inflammatory cytokine secretion, mitigated mTOR signaling, reduced IDO levels and activity, concomitant with enhanced AMPK activity and Sirtuin1 levels [Figs 6 and 7]. DCs and macrophages obtained from AMPK1α-/- mice expressed very high basal levels of pro-inflammatory cytokines such as TNFα suggesting the importance of AMPK signaling in balancing M1 polarization^48^. Further, tryptophan insufficiency signal induced by high IDO in M1 polarized states fires nutrient-sensing mTOR signaling which shifts M1 dependence on fast ATP generating pathways such as glycolysis^27^. MSCs foster a bioenergetic shift at the M1-MSC interface by rescuing tryptophan insufficiency [Fig. 7C], reducing glucose uptake [Figs 6 and 7d,e] and inducing energy conserving AMPK signaling pathways [Fig. 6b,c].

Since PGE_2_ levels were enhanced in the MSC secretome upon treatment with IFNγ, (Supplementary Figure 4) and PGE_2_ signaling has been shown to influence metabolic programs at the tumor cell-stromal interface^49^ we explored the relevance of COX2-generated PGE_2_ from MSCs in directing M1 to M2 bio-energetic shifts taking lessons from the tumor microenvironment. Abrogation of PGE_2_ signaling inputs from MSC secretome at the macrophage-MSC interface enhanced pro-inflammatory cytokine secretion from M1 macrophages over and above the control M1 levels and also rescued IDO activity, glucose uptake and down-modulated *CPT1*α and energy conserving AMPK activity/SIRTUIN 1 suggesting a key mechanistic role for MSC secreted PGE2 in the M1-M2 bioenergetics shift [Fig. 7]. PGE_2_ signaling has been recently implicated in facilitating wound healing in mice through recruitment of M2 macrophages at the wound site in concurrence with observations discussed in this study^50^.

In non-healing wounds, unresolved inflammation instructed by inflammatory macrophages has been reported to challenge endothelial cell survival and neo-angiogenesis^34^. Thus, we further tested the relevance of MSC mediated functional shifts in M1 and M2 macrophages in a M1 mediated endothelial cell injury model (Fig. 8). MSC-primed M1 cells failed to cause endothelial injury, whereas MSC-primed M2 cells facilitated higher endothelial proliferation further substantiating MSC-mediated functional shifts (Fig. 8 and Table 1). These shifts could be the actual reason for better healing responses observed upon allogeneic MSC transplantation in wound models. The data presented in the study would be a stepping stone to resolve these questions in animal models, where vasculopathy is ameliorated upon MSC transplantation.

**Figure 9:**
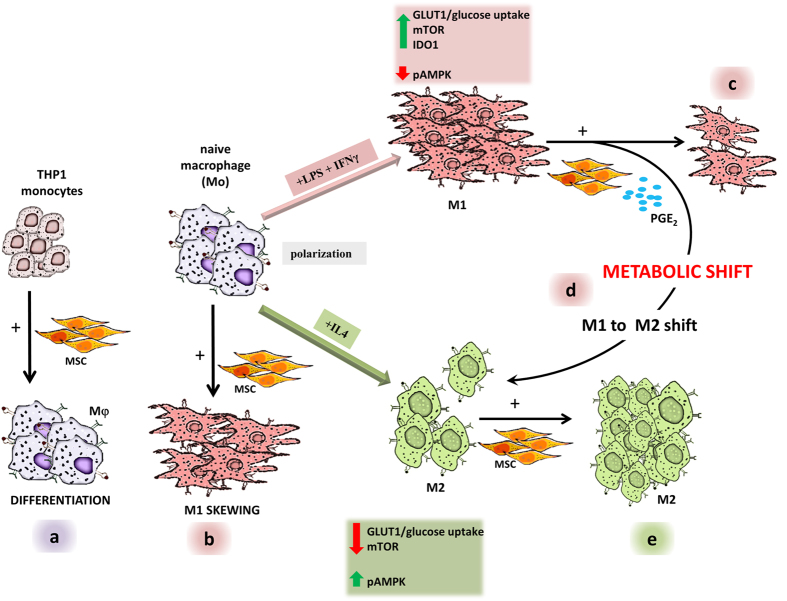
Mechanistic insights into activation state based manipulation of Macrophage plasticity by MSCs. MSCs facilitate (**a**) monocyte to macrophage transition, (**b**) polarize naïve macrophages to an inflammatory M1 activation module and (**e**) enhance M2 activation. MSCs further (**c**) attenuate inflammatory M1 macrophages, (**d**) shifting them to an anti-inflammatory M2 activation state by instructing metabolic shifts in a PGE_2_ dependent manner.

In depth analysis of observations from the present study indicate a pro-active interaction between MSCs and macrophages, wherein, MSCs can shift the balance of M1/M2 polarization fulcrum either towards an inflammatory M1 or a tissue re-modeling, debris scavenging, M2 phenotype based on the activation status of the macrophage upon MSC interaction (Summarized in Fig. 9). MSCs shift “naïve macrophages” to an inflammatory macrophage module without enhancing their APC function; this could be beneficial in an infection scenario, where innate microbicidal immunity would be facilitated without erroneous T cell activation. However, MSCs attenuated already activated pro-inflammatory M1 macrophages by shifting them to a M2 state setting the premise for injury resolution in hyper-inflammatory milieus. When interfaced with M2 activated macrophages, MSCs further enhance the M2 activation threshold. Excessive M2 activation by MSC could prove detrimental in conditions of fibrosis where exaggerated tissue remodeling could prevent optimal repair. In conclusion, a macrophage activation threshold based model for manipulation of macrophage states by MSCs proposed in this study warrants a close look at the polarization status of macrophages in different disease platforms before clinical extrapolation of MSC based benefits in different pathophysiological contexts. Further, we propose a mechanistic role for bioactive factors such as PGE_2_ in the induced MSC secretome in impacting metabolic frameworks of differentially polarized macrophages and instructing M1-M2 polarization shifts [Summarized in Fig. 9].

## Material and Methods

### Cell culture

Human bone marrow mesenchymal stem cells were isolated based on plastic adherence and characterized by flow cytometry and tri-lineage differentiation as per ISCT guidelines according to methods described in previous studies from the lab^31^,^51^.

MSCs between passages 3–6 were used for all the experiments. Bone marrow was obtained with written informed consent from the donor after obtaining prior approval from Ethics committee of Manipal hospitals and Manipal heart foundation (Protocol no: MHB/SCR/010). All experimental protocols described in the study were approved by Ethics committee of Manipal Hospital, Bangalore (Protocol no: MHB/SCR/020). All methods described in the study were carried out in accordance with the approved guidelines.

Human monocytic cell line, THP-1, which has been described previously to resemble human native monocyte and human naïve macrophages (Mφ) upon differentiation, was used to establish the model for human Monocyte-Macrophage differentiation and polarization^52^,^53^. THP-1 cells were grown in RPMI1640 (GIBCO) media containing 10% Fetal bovine serum (FBS) (Thermo), 1 mM Sodium Pyruvate (GIBCO) and 2 mM L-Glutamine (GIBCO) with 1X Anti-anti (GIBCO Life Technologies). THP-1 cells were incubated with 20 nM Phorbol 12-myristate 13-acetate (PMA) for 48 h to differentiate them to macrophages (Mφ). Naïve Mφs were further polarized to inflammatory M1 state by culturing them for 48 h in complete media containing 15 ng/ml of IFNγ and 100 ng/ml of Lipopolysaccharide (LPS). Alternative activation of Mφs to M2 like phenotype was achieved by treatment of THP-1 cells with 20 nM PMA for 6 h and further activation with 10 ng/ml of IL-4 for 24 h in complete medium.

Primary human macrophage cultures were established by treating peripheral blood monocytes with 50 ng/ml of M-CSF (Biolegend) in RPMI1640 complete media for 7 days. Naïve macrophages (Mo) so obtained were further stimulated with 50 ng/ml of LPS and 10 ng/ml of human IFNγ (Invitrogen) for 48 h to polarize them to inflammatory M1 macrophages. Alternate activation of macrophages to M2 polarized state was achieved by treating Mo cells with 20 ng/ml of human IL-4 (Chemicon) for 48 h [protocol sourced from ref. 54].

Human Umbilical vein endothelial cells (HUVECs) were grown on Hu Fibronectin (10 μg/ml) coated plates at a constant seeding density of 2.5 × 10^3^ cells/cm^2^ in Hu Endothelial SFM (GIBCO) supplemented with 1% FBS (HyClone, Australian), 40 μg/ml Endothelial growth factor (SIGMA), 3 ng/ml bFGF (SIGMA) and 100 μg/ml Heparin Sodium (SIGMA).

### MSC/Macrophage co-cultures

Influence of MSCs on monocyte to Mφ differentiation or activation to M1 and M2 functional states were evaluated by culturing both the cell types together in 6 well culture plates (FALCON, CORNING) physically separated by cell-culture inserts (0.4 microns, Millipore) for mRNA analysis, phagocytic assays and microbial clearance assays. 3.5 × 10^5^ THP-1 cells were used for differentiation experiments and 3.5 × 10^4^ MSCs were seeded on culture inserts in co-culture conditions. The ratio was determined based on the ratios of MSCs causing immune-suppression in MLRs as previously reported by our group^31^. For flow-cytometry based evaluation of surface markers on Monocytes, Mφ or M1 and M2 cells, MSCs were differentially labeled with PKH26 red fluorescent cell linker kit (SIGMA) or CFSE (5(6)-carboxyfluorescein diacetate N-succinimidyl (SIGMA), depending on the labeled antibodies used for cell surface marker analysis and gated out from monocytes or macrophages before analysis.

Similar ratios of MSCs and primary macrophages as established in the THP-1 model was used in MSC-human primary macrophage co-cultures. MSCs were co-cultured with either M-CSF differentiated naïve macrophages for 30 hours (referred to as Mo in the manuscript) or were alternatively co-cultured with M1 or M2 activated macrophages for 30 h post 18 h activation with either M1 skewing or M2 skewing stimuli as described above. Co-culture supernatants were collected to quantitate PGE_2_ levels, IDO activity assessment and cytokine analysis. Macrophages grown in 6 well culture plates physically separated by a 0.4 micron culture inserts (on which MSCs were seeded) were harvested for mRNA, protein analysis by immuno-blotting or flow cytometry assessment of specific cell surface antigens.

### Phagocytosis assays

THP-1 cells or PMA differentiated THP-1 cells (Mφ) were cultured in RPMI 1640 complete medium and incubated with 1 mg/ml of 0.0033 μm Dextran-FITC beads (20,000 Daltons) (SIGMA) for 2 h at 37 °C and evaluated for cellular uptake by flow cytometry. Specificity of the phagocytic uptake was ensured by pre-treatment with Cytochalasin-D (10 μg/ml) for 2 h prior to Dextran-FITC bead incubation. Extrinsic surface bound fluorescence was quenched by adding trypan blue before acquisition to quantitate only cellular fluorescence post bead uptake. The phagocytic index was calculated by subtracting the % fluorescent cells with % fluorescence obtained from cells pretreated with Cytochalasin D by flow cytometry.

### Analysis of intracellular ROS and RNI

To evaluate increase in intracellular reactive oxygen species (ROS), cells cultured in basal medium were incubated with 3 μM of redox sensitive agent, DCFH-DA (2′,7′-Dichloro-dihydro-fluorescin diacetate (SIGMA) for 30 minutes at 37 °C in dark and intracellular fluorescence was quantitated by flow cytometry. DCFH-DA fluorescence beyond that generated by intrinsic ROS levels in MO or Mφ was evaluated under MSC co-culture and *Salmonella enterica* 12023 infection conditions after appropriate gating to calculate the relative increase in ROS levels.

NO_2_^−^ levels were measured by modified Griess reaction. 100 μl of Macrophage culture supernatants, diluted or neat, were transferred to 96 well plates in triplicates and equal volume of Griess reagent (SIGMA) was added to the plates and incubated in dark for 15 minutes at 37 °C and absorbance at 540 nm (*A*_540_) was compared to a NaNO_2_ (SIGMA) standard curve to calculate Nitric oxide (NO) levels. RPMI1640 without phenol red media was used for blanking.

### Microbicidal assay

GFP-tagged *Salmonella enterica* 12023 was cultured till they reached logarithmic growth and were opsonized with 5% pooled serum for 20 minutes before incubation with PMA differentiated Mφs. Mφs cultured in antibiotic free medium were incubated with opsonized bacteria for different time intervals at 37 °C at a MOI of 5 as previously optimized in the lab. Following addition of bacteria the plates were pulse centrifuged to allow them to settle onto the adherent macrophages. Post-incubation extracellular bacteria were thoroughly washed with DPBS several times and Mφs were lysed with 1% Triton-X100 (SIGMA). The lysate was collected and evaluated for bacterial count and colony forming ability by pour plate enumeration method at different time intervals of incubation to establish bacterial clearance as previously described^55^.

### Western blotting

Macrophages were washed with Phosphate buffered Saline (PBS) and lysed with ice-cold RIPA buffer containing protease inhibitor cocktail III (CALBIOCHEM) on ice for 30 minutes with intermittent vortexing. Protein in the whole cell extract obtained after collecting the supernatant post-centrifugation at 13000 g for 20 min was separated by a 10–12% SDS-PAGE and transferred to PVDF membrane (MILLIPORE). For detection of total/phosphorylated mTOR by immunoblotting, 8% SDS-PAGE was performed followed by wet transfer [110 mA; 3.5 h; Tris-glycine with 10% methanol and 0.1% SDS; BIOBEE TECH Electro blotting wet transfer system] onto a PVDF membrane.

Post-transfer, PVDF membranes were incubated overnight with either anti-Actin (SantaCruz), anti-NFkB (Millipore), anti-IkBα (Millipore), anti-SOD2 (Millipore), anti-DC SIGN (R & D), anti-IDO1 (chemicon), anti-Sirtuin1 (Cell Signaling Technologies), anti-mTOR/anti phospho-mTOR (Ser2448) (Cell signaling technologies), anti-AMPKα/anti-phospho-AMPKα (Thr172) (Cell Signaling technologies) antibodies. TGM2 monoclonal antibody used in the study was developed by Gail V.W. Johnson and obtained from the Developmental Studies Hybridoma Bank, created by the NICHD of the NIH and maintained at The University of Iowa, Department of Biology, Iowa City, IA 52242.

Subsequently, membranes were probed with appropriate HRP-conjugated secondary antibodies: HRP-goat anti-rabbit, HRP- goat anti mouse or HRP-donkey anti goat secondary antibodies for 1 h. After exhaustive washing with TBST (Tris-buffered Saline with 0.1% Tween 20), specific proteins were detected by 3,3′,5,5′ TETRAMETHYLBENZIDINE (TMB). Images were captured using a Flour Chem FC2 imager (Alpha Innotech). Alternatively, in few cases, signals were acquired using Optiblot ECL Detect Kit [Abcam] on ImageQuant LAS 4000 [GE Health Care Life Sciences] and densitometry analysis was performed using Image J software.

### Enzyme-linked Immunosobent Assay (ELISA)

Secreted protein levels of TNFα, IL-6, IL-10 and IL-12β was quantitated in culture supernatants of control and MSC co-cultured cells using specific cytokine ELISA kits (OptEIA Human ELISA sets; BD Biosciences) as per manufacturer’s instructions. OD was measured using a VICTOR3^TM^ Multi-label plate reader (Perkin Elmer) and the cytokine amounts were estimated from a standard curve deduced from specific cytokine standards.

### Real-time Polymerase chain reaction

Total RNA was isolated using TRI reagent (SIGMA), cDNA was synthesized using RevertAid First Strand cDNA synthesis kit (Thermo). Expression of specific genes was evaluated by qPCR using KAPA SYBR FAST qPCR kit (KAPA BioSystems) on an ABI 7500 qPCR machine using specific primers (Supplementary Table 1). The relative expression levels of each mRNA were normalized to *GAPDH* and ΔC_t_ was calculated. ΔΔC_t_ was calculated after substracting ΔC_t_ of test and ΔC_t_ of the calibrator (untreated monocytes). The fold increase depicted as RQ in the panels was calculated using the formula 2^−ΔΔCt^.

### Flow cytometry

Cell surface markers were analyzed using specific florescence conjugated and non-conjugated antibodies. Non-specific florescence was attuned using appropriate Isotype controls. For monocyte and macrophage specific cell surface antigen expression, mouse anti-human- CD68 FITC, CD14 PE, CD11b APC, HLA-ABC FITC, HLA-DR FITC, CD80 FITC, CD86 PE, CD50 FITC, CD54 PE, CD163 PE, CD205 PE and CD206 APC antibody conjugates from BD Pharmingen; CX3CR1 FITC from R&D Systems were used. To prevent non-specific binding of antibodies to macrophage/monocyte lineage cells, cells were pre-incubated with buffer containing 2 mM EDTA, 0.5% FBS. DPBS and 10 μl of FcR blocker (Miltenyi Biotec) for 15 min at 4 °C. Cells were then washed with DPBS and stained in antibody containing FACS buffer (DPBS containing 1% FBS and 0.01% sodium azide) on ice for 1 hour. For indirect staining, cells were further washed and treated with appropriate secondary antibody for 30 minutes on ice. Stained cells were fixed with 1% Para Formaldehyde and stored at 4 °C till further analysis. Cells were acquired on a BD FACS CALIBUR and the data was analyzed using the Cell Quest Pro software. % positivity was calculated for each surface antigen after gating with respect to relevant isotype control antibody.

### IDO activity assay

IDO activity, represented as concentration of kynurenine produced, was analyzed by a spectrophotometric assay reported earlier^31^. Cell-free spent culture media were harvested from primary macrophage or macrophage/MSC co-cultures and used immediately for assay. To 120 μl of spent medium, 60 μl of 30% trichloroacetic acid [Sigma Aldrich] was added, mixed thoroughly and centrifuged at 8000 g for 5 min at room temperature. From the clarified supernatant, 85 μl was transferred immediately to 96-well plates and 85 μl of 1% Ehrlich reagent prepared in glacial acetic acid [Sigma Aldrich] was added and incubated for 10 min at room temperature. Absorbance was read at 490 nm on a multimode plate reader [Ensight^TM^, Perkin Elmer]. Concentrations were determined against a kynurenine standard [Sigma].

### shRNA-mediated knockdown of *COX2* in MSCs

For generating stable *COX2-*knockdown MSCs, shRNA construct to human *COX2* was purchased [SHCLNG-NM_000963 ; Clone-ID: TRCN0000045533; Sequence: CCGGGCTGAATTTAACACCCTCTATCTCGAGATAGAGGGTGTTAAATTCAGCTTTTTG] from Sigma. Lentiviral particles were prepared using the packaging plasmid psPAX2 [Addgene, Cambridge MA] and envelope-encoding plasmid pMD2.G [Addgene, Cambridge MA] from packaging cell line, 293T. Transfections were performed using X-tremeGENE HP DNA transfection reagent [Roche, Sigma Aldrich]. 48 h and 72 h after transfection, supernatants containing packaged viral particles were concentrated using [Amicon Ultra-4 Centrifugal filter units, Ultracel, Merck Millipore] and used for infecting 75% confluent MSCs [at passage 4]. pLKO containing scrambled shRNA [Addgene, Cambrdige MA] was used as vector control. Infected cells, once confluent, were selected with puromycin [1 ug/ml; ThermoFisher Scientific] for 48 h before being used in co-culture set ups. Knockdown of *Cox2* in MSCs was authenticated by reduction in secreted PGE_2_ levels in IFNγ induced MSCs (Supplementary Figure 4).

### PGE_2_ Quantification

PGE_2_ ELISA was performed with fresh culture supernatants from MSC-macrophage co-cultures using a PGE_2_ forward sequential competitive ELISA kit (R&D Systems; USA) as per manufacturer’s instructions. Absorbance was read at 450 nm with a wavelength correction of 540 nm on an EnSight multimode reader (Perkin Elmer).

### Analysis of Glucose uptake

Following co-culture of primary macrophages with MSC as discussed above, cell culture inserts containing MSCs were removed and macrophages in the well were washed with DPBS to remove any traces of FBS and glucose. Macrophages were then cultured in glucose/serum free RPMI1640 media for 2 hours. To assess glucose uptake in macrophages 25 μM of 2-NBDG (Thermo Fisher) was added to the above media and incubated for additional 20 min. The reaction was stopped by adding cold FBS and chilling the cells in ice. Cells were are then taken for flow cytometry analysis.

### Cell cycle analysis

M1 or M2 activation was set up as discussed above in the presence or absence of MSCs for 48 h on cell culture inserts (MILLIPORE). Post-priming of M1 and M2 macrophages with MSCs, the inserts were transferred on to growing HUVEC cultures in 6 well plates and cultured together for 72 h. HUVECs were trypsinized and stained with cold buffer containing 0.1% Sodium citrate buffer (SIGMA) with 0.1% NP-40 (SIGMA), 0.05 mg/ml RNaseA (SIGMA) and 0.05 mg/ml Propidium Iodide (SIGMA) for 15 minutes on ice. Subsequently cells were analyzed for cell cycle by evaluating the DNA content post-acquisition on a BD FACS CALIBUR^56^.

### Statistical analysis

In almost all experiments values represent mean values of at least three independent experiments with standard error mean (SEM) unless otherwise stated. Each experiment was performed in triplicates with independent bone marrow mesenchymal stem cell isolates between passages 3–6. Each data between different treatment conditions and test samples were compared by using student’s t-test. In all experiments comparisons were made amongst two groups at a time. A p value < 0.05 was considered statistically significant (*), p value < 0.01 and p value < 0.001 is considered very significant (**) and extremely significant (***) respectively.

## Additional Information

**How to cite this article**: Vasandan, A. B. *et al*. Human Mesenchymal stem cells program macrophage plasticity by altering their metabolic status *via* a PGE_2_-dependent mechanism. *Sci. Rep.* **6**, 38308; doi: 10.1038/srep38308 (2016).

**Publisher’s note:** Springer Nature remains neutral with regard to jurisdictional claims in published maps and institutional affiliations.

## Supplementary Material

Supplementary Information

## Figures and Tables

**Table 1:** Cell cycle analysis of HUVECs co-cultured with MSC primed or unprimed M1/M2 macrophages (+).

	Control	+M1	+MSC-primed M1	+M2	+MSC primed M2
Sub Go	1 ± 0.01	17.3 ± 1.3	1.7 ± 0.1**	1.8 ± 0.03	1.5 ± 0.13
Go/G1	67.7 ± 7.2	25.3 ± 5.8	69 ± 9.3*	68.3 ± 8.7	60.9 ± 2.3
S	13.4 ± 1.7	20.4 ± 6.3	12.9 ± 4.6	11.9 ± 4.3	20.3 ± 1.3*
G2M	15.6 ± 1.1	30.4 ± 10.9	14.2 ± 2.5	15.3 ± 1.5	17.9 ± 1.4

Control refers to untreated HUVEC cells. Values indicate % positive cells in each phase of the cell cycle ± SEM (n ≥ 3). p values indicate comparisons between cycling parameters of HUVECs co-cultured with macrophages *versus* HUVECs co-cultured with MSC primed macrophages.
