# Supplementary material for: Human Mesenchymal stem cells program macrophage plasticity by altering their metabolic status via a PGE2-dependent mechanism
Source: Sci Rep. 2016 Dec 2;6:38308. doi: 10.1038/srep38308 (PMC5133610; doi:10.1038/srep38308)
Supplement: Supplementary Information [file srep38308-s1.pdf]

Human Mesenchymal stem cells program macrophage plasticity by altering their metabolic status *via* a PGE_2-dependent mechanism

Anoop Babu Vasandan[1], Sowmya Jahnavi[1], Chandanala Shashank[1], Priya Prasad[1], Anujith Kumar[1], S. Jyothi Prasanna[1*]

[1] School of Regenerative Medicine, Manipal University, Bangalore-560065, India

Email: ***** jyothi.prasanna@manipal.edu

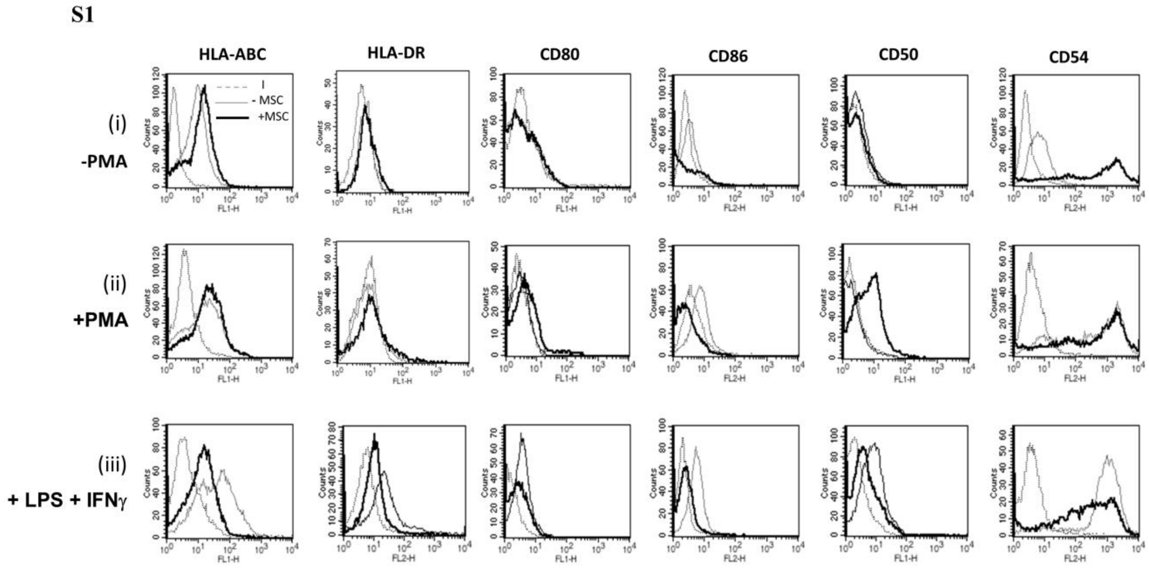

Supplementary figure 1: MSCs influence surface expression of key co-stimulatory and immune-adhesive ligands on PMA differentiated and M1 polarized human THP-1 Cells. Flow cytometry of surface antigens associated with APC-capacity of THP1 (i) PMA differentiated THP1 Mφ (ii) M1 polarized THP1 cells (iii). Histogram overlays depict relevant isotype control (I; black dotted line), Thick black line and thin black line indicate surface expression of the specific ligand with and without MSCs respectively. Histograms are representative of three independent experiments.

S2

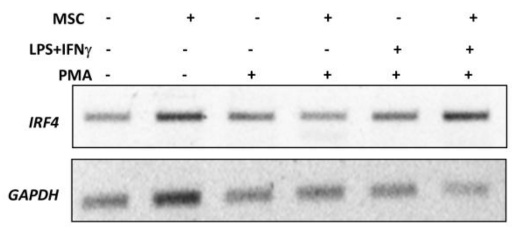

Supplementary figure 2:*IRF4* transcript levels in MSC-macrophage co-cultures evaluated on a 1% agarose gel. PCR was run for 20 cycles for *GAPDH* and 25 cycles for *IRF4*.

S3

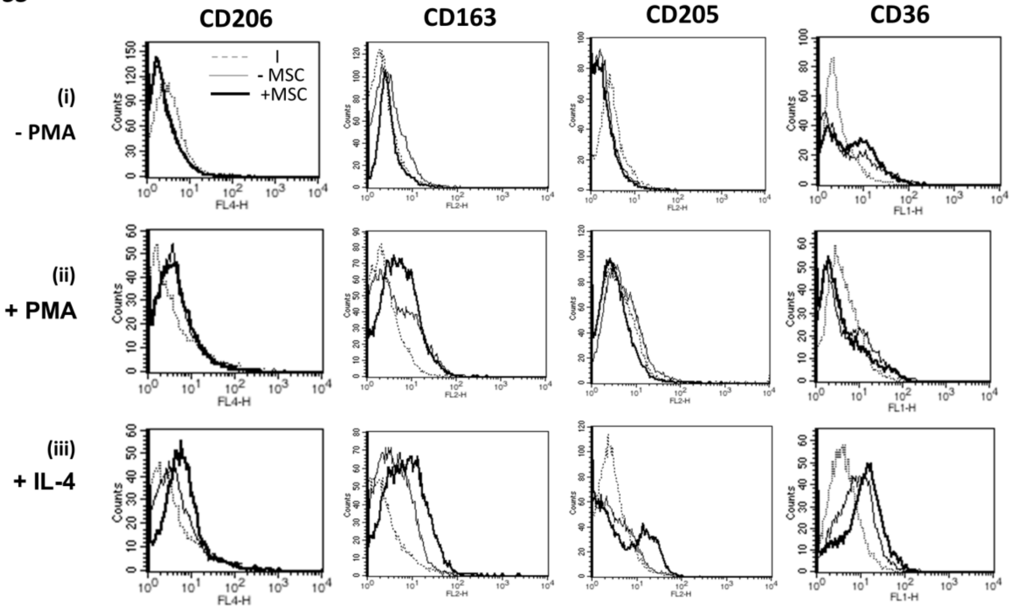

Supplementary figure 3: MSCs influence surface expression of key scavenger receptors on PMA differentiated and M2 polarized human THP-1 Cells. Flow cytometry of surface antigens associated with scavenger receptors on THP1 (i) PMA differentiated THP1 Mφ (ii) M2 polarized THP1 cells (iii). Histogram overlays depict relevant isotype control (I; black dotted line), Thick black line and thin black line indicate surface expression of the specific ligand with and without MSCs respectively. Histograms are representative of three independent experiments.

S4

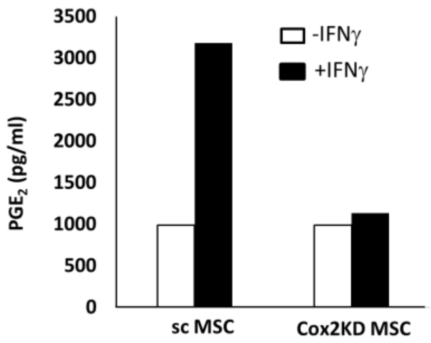

Supplementary figure 4: *Cox2* KDN resulted in decrease in PGE_2 secretion in IFNγ primed MSC. MSCs were primed with 50 ng/ml of IFNγ for 48 hours and PGE_2 was analyzed in the culture supernatants as described in material and methods.

Supplementary Table 1

Primers used for real time PCR analysis

GENE	FORWARD PRIMER (5'-3')	REVERSE PRIMER (5'-3')
ALOX15	CTTAAGGACGACGCCTGGTT	AGTTTCCCCACCGGTACAAC
BAX	GCCGTGGACACAGACTCC	AAGTAGAAAAGGGCGACAACC
BCL2	CACCTGTGGTCCACCTGAC	ACGCTCTCCACACACATGAC
CCL13	CAGATGCACTCAACGTCCCA	TGGTTCTGAAGATGACAGCCTT
CCR7	GGCTGGTCGTGTTGACCTAT	ACGTAGCGGTCAATGCTGAT
CD11b	CCATAGCCAGCGGATAGCAG	CTTGCCACTTCCCTGGGATT
CD14	CACAGTGGGAGCCGCACAGG	ACGTTGCGTAGGCGCAAGCT
CD206/MRC1	CCAGGGCGAAAGCCAGGGTG	GTCGTGGGCTTCGGTGGGTG
CD36	ACTGAGGACTGCAGTGTAGGA	ACAAGCTCTGGTTCTTATTCACA
CD68	TGGGTGGGATCATCTCCAGT	CCCCTGGGCTGCCAGTA
CD86	TGGAGAGGGAAGAGAGTGAACA	GCCCATAAGTGTGCTCTGAA
CPT1α	CCAGACGAAGAACGTGGTCA	ATCTTGCCGTGCTCAGTGAA
DC-SIGN	GAACTGGCACGACTCCATCA	GTTGGGCTCTCCTCTGTTCC
GAPDH	CGACCACTTTGTCAAGCTCA	AGGGGTCTACATGGCAACTG
HK2	GATTTCACCAAGCGTGGACT	CCACACCCACTGTCACTTTG
IDO	CCTGAGGAGCTACCATCTGC	TCAGTGCCTCCAGTTCCTTT
IL-10	AAGCCTGACCACGCTTTCTA	ATGAAGTGGTTGGGGAATGA
IL-12β	CCAAGGGGTGACGTGCGGAG	GGTGGGTCAGGTTTGATGATGTCCC
IL-6/IFNβ2	GAACTCCTTCTCCACAAGCG	TTTTCTGCCAGTGCCTCTTT

GENE	FORWARD PRIMER (5'-3')	REVERSE PRIMER (5'-3')
IRF4	CTCTTCAAGGCTTGGGCACT	AGGGTAAGGCGTTGTCATGG
IRF5	TAGAGGCTACCCAGGAGCAA	GCCCACTCCAGAACACCTTA
MSR1	TTCAACGCAGGAATGTGTCA	AGCTGTCATTGAGCGAGCAT
p53	GAAGACCCAGGTCCAGATGA	TTCTGGGAAGGGACAGAAGA
PCNA	CCTGGCCATGGGCGTGAACC	AGCGCCAAGGTATCCGCGTT
PHOX p22	GGGCCATGTGGGCCAACGAA	CACGCCCGCCACAATGGAGT
PHOX p47	CGCAGGTCGTCCATCCGCAA	CGGACGCTGTTGCGGCGATA
PHOX p91	TGTGTGAATGCCCGAGTCAA	ATGGATGGCAAGGCCAATGA
SIRT1	CACACTTTTAGACCAAGCAGCTAA	TTTCTCCCCAGTACTAGAACCAAC
SOD2	GGAACAACAGGCCTTATTCCAC	GAGCTTAACATACTCAGCATAACGA
TGFβ1	CCTACATTTGGAGCCTGGAC	TGTCCTTAAATACAGCCCCC
TGM2	GCCCACATCACCAACAACAC	GTAGAGGTCCCTCTCAGCCA
TNFα	TGGCCAATGGCGTGGAGCTG	GTAGGAGACGGCGATGCGGC

Cycling conditions (a) Real time PCRs: Initial denaturation at 50°C at 2 min and 95 °C for 10 min. cycling at 40 cycles: Denaturation at 95 °C for 15 sec and Annealing/extension at 60 °C for 1 min. **(b)** RT-PCR: Initial denaturation at 95 °C for 5 min; Denaturation at 95 °C for 30 sec, 61 °C for 45 sec and 72 °C for 30sec; final extension 72 °C for 10min.
